# Accuracy of Deep Learning Echocardiographic View Classification in Patients with Congenital or Structural Heart Disease: Importance of Specific Datasets

**DOI:** 10.3390/jcm11030690

**Published:** 2022-01-28

**Authors:** Felix K. Wegner, Maria L. Benesch Vidal, Philipp Niehues, Kevin Willy, Robert M. Radke, Philipp D. Garthe, Lars Eckardt, Helmut Baumgartner, Gerhard-Paul Diller, Stefan Orwat

**Affiliations:** 1 Department of Cardiology II—Electrophysiology, University Hospital Muenster, Albert-Schweitzer-Campus 1, 48149 Muenster, Germany; philipp.niehues@ukmuenster.de (P.N.); kevin.willy@ukmuenster.de (K.W.); lars.eckardt@ukmuenster.de (L.E.); 2 Department of Cardiology III—Adult Congenital and Valvular Heart Disease, University Hospital Muenster, Albert-Schweitzer-Campus 1, 48149 Muenster, Germany; maria.beneschvidal@ukmuenster.de (M.L.B.V.); robert.radke@ukmuenster.de (R.M.R.); philipp.garthe@ukmuenster.de (P.D.G.); helmut.baumgartner@ukmuenster.de (H.B.); gerhard.diller@ukmuenster.de (G.-P.D.); stefan.orwat@ukmuenster.de (S.O.)

**Keywords:** deep learning, neural network, structural heart disease, congenital heart disease, echocardiography

## Abstract

Introduction: Automated echocardiography image interpretation has the potential to transform clinical practice. However, neural networks developed in general cohorts may underperform in the setting of altered cardiac anatomy. Methods: Consecutive echocardiographic studies of patients with congenital or structural heart disease (C/SHD) were used to validate an existing convolutional neural network trained on 14,035 echocardiograms for automated view classification. In addition, a new convolutional neural network for view classification was trained and tested specifically in patients with C/SHD. Results: Overall, 9793 imaging files from 262 patients with C/SHD (mean age 49 years, 60% male) and 62 normal controls (mean age 45 years, 50.0% male) were included. Congenital diagnoses included among others, tetralogy of Fallot (30), Ebstein anomaly (18) and transposition of the great arteries (TGA, 48). Assessing correct view classification based on 284,250 individual frames revealed that the non-congenital model had an overall accuracy of 48.3% for correct view classification in patients with C/SHD compared to 66.7% in patients without cardiac disease. Our newly trained convolutional network for echocardiographic view detection based on over 139,910 frames and tested on 35,614 frames from C/SHD patients achieved an accuracy of 76.1% in detecting the correct echocardiographic view. Conclusions: The current study is the first to validate view classification by neural networks in C/SHD patients. While generic models have acceptable accuracy in general cardiology patients, the quality of image classification is only modest in patients with C/SHD. In contrast, our model trained in C/SHD achieved a considerably increased accuracy in this particular cohort.

## 1. Introduction

Transthoracic echocardiography represents one of the main diagnostic modalities in modern clinical cardiology with a broad range of uses and indications [1]. While traditionally manually interpreted by cardiologists, novel computer-based technologies allow deep neural networks (DNN) to increasingly assist in the assessment of acquired images [2,3,4,5]. This may especially aid sonographers with infrequent echocardiography practice [6,7] and expand the conduction of echocardiography to settings thought to be outside the scope of this modality, such as primary care or medical care in remote areas [8].

As the necessary first step in the automated interpretation of echocardiography images, views need to be correctly identified. Similar to other cardiac imaging modalities such as magnetic resonance imaging (MRI) or computer tomography (CT), echocardiography studies require a multitude of views depicting the cardiac structures. Theoretically, an infinite number of different views are possible, but 27 of these have been identified as the views that should be taken during the performance of a comprehensive transthoracic echocardiography examination, with a core 15–20 views employed in almost all studies depending on the diagnostic question [1]. Importantly, by dissecting cardiac chambers and valves at different angles through a differently rotated two-dimensional echocardiography probe, operators depict specific parts of the structure of interest [9] with some anatomic abnormalities only detectable in a few views. Conversely, in the important question of regional wall motion abnormalities, the information of up to seven views (apical two-, three- and four-chamber, parasternal short axis at the level of the mitral valve, papillary muscles and apex and parasternal long axis) needs to be combined to analyze all segments of the left ventricle [10]. Therefore, misidentification on automated analysis may impede patient care and could potentially delay a correct diagnosis [11].

In this regard, a recent study by Zhang et al. [12] proposed and validated a convolutional neural network to assess echocardiographic images and classify them according to the depicted echocardiographic view. While the authors report a high accuracy in the identification of the correct echocardiographic view, it is unclear whether these findings can be applied to patients with congenital or structural heart disease (C/SHD). The present study was, therefore, conducted to assess the performance of a DNN trained and validated in a cohort of general cardiology patients concerning its performance in classifying views depicting structural and congenital heart disease and compare it to a DNN specifically trained in C/SHD.

## 2. Methods

The present study was conducted according to the Declaration of Helsinki and its later amendments. The project was approved by the local ethics committee (Approval number 2020-751-f-S).

### 2.1. Imaging Database

**Figure 1:**
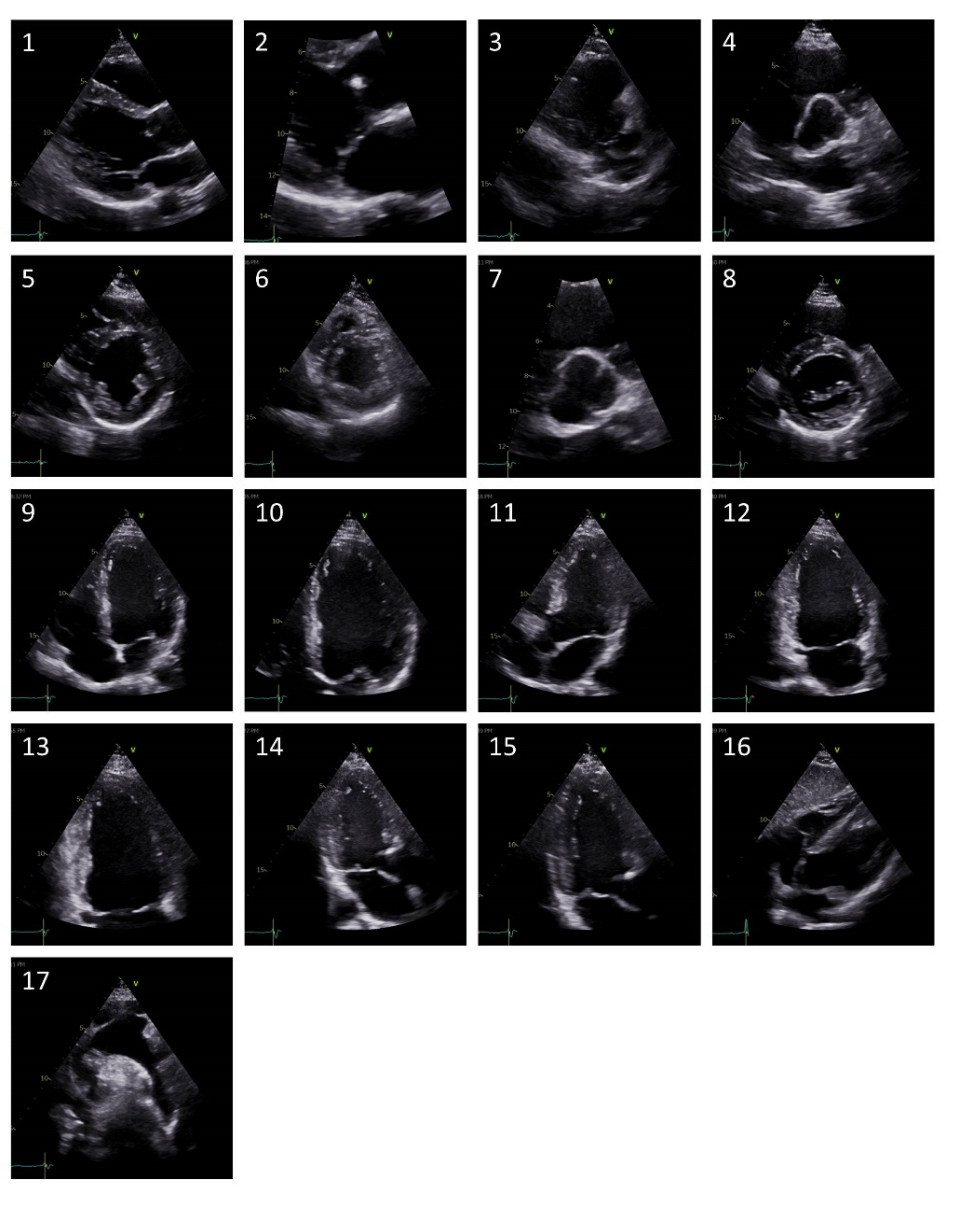
Representative images of the included seventeen separate echocardiographic views. (**1**) = Parasternal long axis (PLAX) left ventricle, (**2**) = PLAX zoomed MV, (**3**) = PLAX RV inflow, (**4**) = Parasternal short axis (PSAX) focus on AV, (**5**) = PSAX papillary muscles, (**6**) = PSAX apex, (**7**) = PSAX zoomed AV, (**8**) = PSAX MV, (**9**) = Apical 4 chamber (A4C), (**10**) = A4C zoomed left ventricle, (**11**) = Apical 5 chamber, (**12**) = Apical 2 chamber (A2C), (**13**) = A2C zoomed left ventricle, (**14**) = Apical 3 chamber (A3C), (**15**) = A3C zoomed left ventricle, (**16**) = Subcostal 4 chamber, (**17**) = Suprasternal aortic arch.

Echocardiograms of patients with congenital and structural heart disease were selected retrospectively from the routine clinical imaging database of the Department of Cardiology III—Adult Congenital and Valvular Heart Disease at the University Hospital Muenster. Echocardiograms were chosen for diversity of underlying disease etiology (see Table 1), comprehensiveness of echocardiographic views and quality of acquired loops. In addition, echocardiograms of patients without a cardiac abnormality were prospectively included according to the aforementioned criteria. The examinations were performed on different echocardiography machines from different vendors (especially GE Vivid E9, Vivid E95, Vivid 7; Philips EPIC 7C, EPIC 7G, iE33). Two-dimensional (2D) echocardiographic studies performed according to current guideline recommendations [1] were anonymized, exported and converted into individual frames in a PNG format for automated analysis. In total, individual frames of 17 separate TTE views were obtained. Figure 1 details the utilized echocardiography views.

### 2.2. Convolutional Neural Networks

The source code and model weights of the DNN trained with a general dataset were obtained from https://bitbucket.org/rahuldeo/echocv (accessed on 20 March 2021) and the training and validation methodology was previously published by Zhang et al. [12]. To summarize, a 13-layer convolutional neural network (VGG 13) was trained with images assigned an individual view label and five-fold cross-validation was used to assess accuracy. Because of a lack of images for some uncommon views in the C/SHD-cohort, we decided to use 17 instead of the original 23 different views. Additionally, for ease of comparison, we assessed single echocardiographic frames individually instead of averaging accuracy across frames of the same image loop.

For the DNN trained with a C/SHD-specific dataset, our echocardiographic dataset was split into a training/validation group (80%) and a test group (20%). Frames from patients of the test group were not used for model training to ensure the external validity of the new model. Image resolution was reduced to 150 × 150 pixels and a greyscale of 256 shades.

During training image augmentation with random rotations (±10°), width and height shifts (10 and 5%, respectively) as well as shears and zoom (up to 10% and 5%, respectively) were applied to the echocardiographic images at run-time. To this end a pre-trained VGG-19 network implemented in Tensorflow/Keras was utilized as described before (https://arxiv.org/abs/1409.1556, accessed on 20 March 2021). For transfer learning weights of the convolutional base were initially frozen and the model was trained for 50 epochs. Subsequently weights from convolutional layer 3, block 4 upwards were unfrozen and the model trained for an additional 80 epochs. This approach was chosen to protect previously learned representations and to ensure the best possible accuracy while avoiding overfitting. This was done by continuously inspecting training and validation accuracy/loss. The model accuracy was quantified as the percentage of correctly classified frames. Training and testing were performed on an Intel i9 platform with GPU support (Nvidia GX 2080Ti). Analyses were performed using RStudio Version 1.4.1717/R-package version 4.1.0. For further information, see the Supplementary Materials.

### 2.3. Statistical Analysis

For the direct comparison of the different DNN’s accuracy, a contingency table of correctly and incorrectly identified views was created and analyzed with the Chi-square test with SPSS Version 27 (IBM Corporation, Somers, NY, USA). Statistical significance was defined as a two-sided alpha level of 0.05 or less.

## 3. Results

### 3.1. Deep Neural Network (DNN) Trained with a General Dataset

Transthoracic echocardiograms of 262 patients with C/SHD were identified for inclusion. Patient characteristics and individual congenital or structural abnormalities of the population are depicted in Table 1. In addition, echocardiography studies of 62 patients (mean age 45 years, 50.0% male) without a cardiac abnormality were identified and included for automated view classification. In total, 9793 TTE loops were included for the patient group with C/SHD. Of these, 8371 loops were acquired on GE ultrasound systems (Vivid 7 or Vivid E95) and 1422 loops were acquired on Philips ultrasound systems (Epiq). For the group with normal cardiac anatomy, 706 loops were included in the analysis. In total, 284,250 individual frames were assessed for view classification by the DNN trained with a general dataset in the present study. Overall, the accuracy of the DNN trained with a general dataset concerning view classification was 48.3% in patients with C/SHD (see Table 2) on a frame by frame basis. The highest accuracy was achieved in the identification of the parasternal long axis (76.5% correct) and the subcostal 4 chamber view (87.7% correct). In contrast, the DNN had a low accuracy in distinguishing the different parasternal short axis views and apical views (see Table 2).

The DNN’s accuracy for view classification was 66.7% overall in patients without a cardiac abnormality (see Table 3). In this group of patients, identification of the parasternal long axis and subcostal 4 chamber view remained very accurate (98.4% and 100%, respectively), but the differentiation between separate parasternal short axis and apical views was higher compared with C/SHD-frames. For example, a parasternal short axis view at the level of the papillary muscles was correctly identified by the DNN in 63.0% of frames depicting C/SHD compared with 79.4% of frames without cardiac abnormality and the apical 4 chamber view was correctly identified in 52.7% of frames with C/SHD versus 77.5% of frames without cardiac abnormality.

### 3.2. DNN Trained with a Congenital or Structural Heart Disease (C/SHD)-Specific Dataset

A new convolutional neural network was independently trained on 139,910 frames depicting C/SHD and subsequently tested on a dataset of 35,614 frames. Table 4 depicts a cross matrix of this DNN’s accuracy in the identification of the 17 utilized echocardiographic views. The overall accuracy across all views was 76.1%. Similar to the DNN trained with a general dataset, the parasternal long axis and subcostal views were distinguished with a high accuracy by the DNN trained with a C/SHD-specific dataset. However, this DNN showed a higher accuracy over the DNN trained with a general dataset in the classification of parasternal short axis and apical views. For example, the DNN trained with a C/SHD-specific dataset was able to detect a parasternal short axis view of the mitral valve with an accuracy of 52% compared to an accuracy of 11.3% by the DNN trained with a general dataset. Additionally, the apical 2-, 3-, 4- and 5-chamber views were able to be distinguished with a very high accuracy by the DNN trained with a C/SHD-specific dataset (80%, 88%, 78% and 91%, respectively) compared to the DNN by Zhang et al. (31.3%, 28.5%, 52.7% and 25.5%, respectively). This resulted in a highly statistically significant difference in the accuracy of the DNN trained with a C/SHD-specific dataset compared with the DNN trained with a general dataset in the view classification of patients with C/SHD (*p* < 0.001).

## 4. Discussion

The present study is the first to directly compare the accuracy of a convolutional neural network developed in general cardiology cohorts with a neural network trained with a C/SHD-specific dataset for echocardiogram view classification. We were able to demonstrate the superiority of a DNN trained with a C/SHD-specific dataset in the identification of echocardiographic views in this distinct group of patients.

Automated view classification is an important part of completely autonomous echocardiography interpretation by deep neural networks [13]. Zhang et al. [12] as well as other working groups [13,14] and our present study document the high accuracy in the identification of echocardiographic views by a DNN trained and validated in a cohort of general cardiology patients when applied to these patients or patients without a cardiac abnormality. However, the present study indicates that this DNN’s precision is considerably decreased in a patient population with underlying congenital or structural heart disease. This is comprehensible from a clinical point of view as patients with a diverse set of congenital and structural heart diseases such as our included patient population display various abnormalities ranging from displaced or abnormally configured valves (Ebstein anomaly) to atypically configured (non-compaction cardiomyopathy) or virtually absent heart chambers (hypoplastic left heart syndrome). Since patients with C/SHD are at an increased risk of morbidity and mortality [15,16], misidentification in automated clinical workflows may impede care in this patient population already at a far greater risk than the general population.

A recent study by Narang et al. [6] evaluated a deep neural network in guiding medical personnel previously untrained in echocardiography to obtain diagnostic TTE images for evaluation of right- and left-ventricular function and the presence of pericardial effusion. While the need for manual acquisition of images and the range of different imaging planes may slow the automation of echocardiography, this demonstrates the profound changes in the practice of echocardiography possible with the utilization of artificial intelligence [17,18]. Although likely increasing efficiency in the diagnosis and treatment of common cardiac conditions such as ischemic cardiomyopathy, comparatively rare disorders such as a congenital heart disease may be underdiagnosed or misclassified by DNNs not trained in these specific conditions. This may be especially important in common echocardiography indications such as the quantification of ejection fraction or pericardial effusion. Further studies should aim to integrate or combine models trained in general cardiology and C/SHD cohorts to avoid possible blind spots concerning uncommon diseases.

While a previous study has reported on convolutional neural networks trained and validated for view classification in patients with atrial or ventricular septal defects [19], the present DNN was trained in a larger and more diverse patient population. Atrial and ventricular septal defects are an important and common congenital malformation, but their presence usually does not substantially alter the overall structure of the cardiac chambers. Conversely, our included patient population contained patients with a variety of congenital and structural heart diseases which may substantially alter the cardiac structures and, therefore, present an independent challenge in view classification for both human interpreters and artificial intelligence.

### Limitations

The present study was conducted as a single-center experiment and echocardiographic studies were gathered from a large tertiary university center for adult congenital heart disease. Therefore, included images may be of greater homogeneity than might be achievable in a community, multi-center setting. However, included images were obtained across different ultrasonography systems by multiple echocardiographers with considerable variation in underlying disease and image quality. Importantly, studies were acquired across the spectrum of inpatient and outpatient indications and a diverse range of underlying disease etiologies. We focused on exploring the importance of disease-specific datasets for accurate view-classification. Alternatively, a systematic exploration of various CNN designs and their impact on the accuracy of view detection could have been investigated. Further work is required to assess whether optimizing the underlying CNN model would improve classification results beyond using disease-specific datasets.

## 5. Conclusions

Automated view classification is an important part of echocardiographic interpretation by deep neural networks. While a convolutional neural network trained in general cardiology patients showed acceptable accuracy in this cohort, echocardiographic views in patients with congenital or structural heart disease were frequently misidentified. In this regard, a convolutional neural network trained specifically in this subset of patients showed a much improved accuracy, highlighting the need for specific neural networks in this important group of patients.

## Figures and Tables

**Table 1:** Characteristics of the patient population with congenital or structural heart disease.

Diagnosis	Number (%)	Mean Age in Years	Male (%)
Ebstein anomaly	18 (7%)	40	14 (78%)
Hypoplastic left heart	3 (1%)	23	2 (67%)
Tricuspid atresia	1 (<1%)	36	1 (100%)
Non-compaction cardiomyopathy	9 (3%)	37	7 (78%)
Transposition of the great arteries (TGA)	48 (18%)	39	25 (52%)
Tetralogy of Fallot	30 (11%)	47	15 (50%)
Incomplete atrio-ventricular septal defect	6 (2%)	39	1 (17%)
Complete atrio-ventricular septal defect	5 (2%)	55	2 (40%)
Double outlet right ventricle	1 (<1%)	50	1 (100%)
Atrial septal defect	7 (3%)	37	4 (57%)
Amyloidosis	23 (9%)	68	20 (87%)
Hypertrophic obstructive cardiomyopathy	7 (3%)	58	3 (43%)
Fabry disease	11 (4%)	60	7 (64%)
Dilatative cardiomyopathy	54 (21%)	58	35 (65%)
Congenitally corrected TGA	34 (13%)	48	18 (53%)
Muscular ventricular septal defect	5 (2%)	42	1 (20%)
All patients	262	49 ± 17	156 (60%)

**Table 2:** Confusion matrix of the general algorithm results in classification of echocardiographic views of patients with CSHD. A2C = Apical 2 chamber, A3C = Apical 3 chamber, A4C = Apical 4 chamber, PLAX = Parasternal long axis, PSAX = Parasternal short axis. Background colors depict strength of classification.

		General Algorithm Interpretation in %																	
**Ground truth**		**1**	**2**	**3**	**4**	**5**	**6**	**7**	**8**	**9**	**10**	**11**	**12**	**13**	**14**	**15**	**16**	**17**	**other**
	PLAX left ventricle (1)	76.5	5.9	0.4	4.8	0.4		0.4	0.2								3.6	0.2	7.9
	PLAX zoomed MV (2)		33.3		33.3												33.3		
	PLAX RV inflow (3)	18.2		40.9	18.2	4.5				9.1							4.5		4.5
	PSAX focus on AV (4)	6.5	0.7		69.9	0.3	0.3	4.794									8.2	0.3	8.9
	PSAX papillary muscles (5)	7.7		2.7	5.4	63.0	0.8	0.3	3.8	0.5	0.3			0.3	0.3		10.2		4.8
	PSAX apex (6)	9.6		0.8	3.2	59.7	3.2		1.6		0.8						11.3	2.4	7.3
	PSAX zoomed AV (7)				40.0			20.0									40.0		
	PSAX MV (8)	20.8	0.4	2.0	7.5	33.8	0.5	0.4	11.3	0.5	1.1	0.2		0.5	0.2		10.7	0.2	10.0
	Apical 4 chamber (9)	1.8	0.2	0.5	3.1	0.3				52.7	1.9	23.7	1.1	2.4	1.1	3.0	2.6		3.3
	A4C zoomed left ventricle (10)	2.8			2.8	4.7		0.5		10.4	62.7	0.5	10.4			0.5	0.9	0.5	3.3
	Apical 5 chamber (11)	2.7			2.7	0.9				20.0	26.4	25.5	1.8	2.7		1.8	4.5		10.9
	Apical 2 chamber (12)	5.6	0.9	0.6	3.8	0.6				4.1	2.8		31.3	30	3.7	4.0	4.4		8.1
	A2C zoomed left ventricle (13)	2.2		3.3	1.1	1.1					15.4		2.2	57.1	1.1	6.6	3.3		6.6
	Apical 3 chamber (14)	14.4	1.6	1.0	5.5	0.3				2.6	2.0	0.7	1.0	1.6	28.5	31.3	1.3		8.8
	A3C zoomed left ventricle (15)				11.1	11.1					11.1	11.1			11.1	44.4			
	Subcostal 4 chamber (16)	0.5			1.0	1.0					1.5		0.5				87.7		7.8
	Suprasternal aortic arch (17)			1.9	17.9	6.0		6.0						1.5			19.4	34.3	13.0

**Table 3:** Confusion matrix of general algorithm results in classification of echocardiographic views of patients without cardiac abnormality. A2C = Apical 2 chamber, A3C = Apical 3 chamber, A4C = Apical 4 chamber, PLAX = Parasternal long axis, PSAX = Parasternal short axis. Background colors depict strength of classification.

		General Algorithm Interpretation in %																	
**Ground truth**		**1**	**2**	**3**	**4**	**5**	**6**	**7**	**8**	**9**	**10**	**11**	**12**	**13**	**14**	**15**	**16**	**17**	**other**
	PLAX left ventricle (1)	98.4	1.6																
	PLAX zoomed MV (2)	33.3	66.6																
	PLAX RV inflow (3)			75.0															25.0
	PSAX focus on AV (4)	19.4		2.8	63.8			8.3									2.8		2.8
	PSAX papillary muscles (5)	7.2	1.0	2.0	1.0	79.4											6.2		3.1
	PSAX apex (6)					94.5	0										5.5		
	PSAX zoomed AV (7)							-											
	PSAX MV (8)	10.6	2.1	2.1	4.3	48.9			19.1								10.6		2.1
	Apical 4 chamber (9)		0.7							77.5	20.5	1.3							
	A4C zoomed left ventricle (10)									16.7	66.7	4.2	4.2	4.2			4.2		
	Apical 5 chamber (11)				14.3					35.7	21.4	28.6							
	Apical 2 chamber (12)												57.1		21.4	21.4			
	A2C zoomed left ventricle (13)	11.1			11.1						22.2		22.2	11.1		11.1			11.1
	Apical 3 chamber (14)	13.3			6.7					6.7			6.7	6.7	33.3	26.7			
	A3C zoomed left ventricle (15)	66.7														33.3			
	Subcostal 4 chamber (16)																100		
	Suprasternal aortic arch (17)	20.0																40.0	40.0

**Table 4:** Confusion matrix of the results of the CSHD-specific algorithm in classification of echocardiographic views of patients with CSHD. A2C = Apical 2 chamber, A3C = Apical 3 chamber, A4C = Apical 4 chamber, CSHD = Congenital or structural heart disease, PLAX = Parasternal long axis, PSAX = Parasternal short axis. Background colors depict strength of classification.

		CSHD-Specific Algorithm Interpretation in %																	
**Ground truth**		**1**	**2**	**3**	**4**	**5**	**6**	**7**	**8**	**9**	**10**	**11**	**12**	**13**	**14**	**15**	**16**	**17**	**other**
	PLAX left ventricle (1)	94		2	1		1			1			1						
	PLAX zoomed MV (2)	94	5				1												
	PLAX RV inflow (3)	1		80	1		9	3		4								2	
	PSAX focus on AV (4)	2		3	88		2			1							3		
	PSAX papillary muscles (5)	1		1		68			26	3	1		1						
	PSAX apex (6)				2	48	6	2	26	6	6			3			6	1	
	PSAX zoomed AV (7)																		
	PSAX MV (8)	1		1		46			52										
	Apical 4 chamber (9)		1		1					91	4	1	1		1				
	A4C zoomed left ventricle (10)									10	69	20	1						
	Apical 5 chamber (11)									21	1	78							
	Apical 2 chamber (12)					6				6			80	5	2		1		
	A2C zoomed left ventricle (13)					3			6	27			32	32					
	Apical 3 chamber (14)					4			3	2	3				88				
	A3C zoomed left ventricle (15)												3		90	7			
	Subcostal 4 chamber (16)																100		
	Suprasternal aortic arch (17)						6										1	93	

## Supplementary Materials

The following supporting information can be downloaded at: https://www.mdpi.com/article/10.3390/jcm11030690/s1. Codes of the generation and training: the VGG19 deep learning network.
