# Supplementary material for: Accuracy of Deep Learning Echocardiographic View Classification in Patients with Congenital or Structural Heart Disease: Importance of Specific Datasets
Source: J Clin Med. 2022 Jan 28;11(3):690. doi: 10.3390/jcm11030690 (PMC8836991; doi:10.3390/jcm11030690)
Supplement: Supplementary file 1 [file jcm-11-00690-s001.zip › jcm-1525811-supplementary.pdf]

__Supplementary Material__

Code template to replicate the generation and training of the VGG19 deep learning network as used in the current paper:

```
# run on:
# R version 3.5.1 and R-package version 4.1.0
# Tensorflow version 1.8
# keras package version 2.1.6
# CUDA version 9.0.176

library(keras)

# Building the network:
conv_base <- application_vgg19(
  weights ="imagenet",
  include_top = FALSE,
  input_shape = c(224, 224, 3)
)

model <- keras_model_sequential() %>%
  conv_base() %>%
  layer_flatten() %>%
  layer_dense(units = 256, activation = 'relu') %>%
  layer_dense(units = 18, activation = "softmax")


model    # output the model
conv_base  #output the vgg19

freeze_weights(conv_base)

model %>% compile(
  loss = "categorical_crossentropy",
  optimizer = optimizer_rmsprop(lr = 2e-5),
  metrics = c("acc")
)

# train_generator and validation_generator to be defined according to local data
structure

history <- model %>% fit_generator(
  train_generator,
  steps_per_epoch = 100,
  epochs = 50,
```

```
  validation_data = validation_generator,
  validation_steps = 50
)

# unfreeze part of the convolutional base

unfreeze_weights(conv_base, from="block4_conv3")
model %>% compile(
  loss = "categorical_crossentropy",
  optimizer = optimizer_rmsprop(lr = 1e-5),
  metrics = c("acc")
)


history <- model %>% fit_generator(
  train_generator,
  steps_per_epoch = 100,
  epochs = 80,
  validation_data = validation_generator,
  validation_steps = 50
)
```

Representation of the overall Model as used:

Layer (type)	Output Shape	Param #
vgg19 (Model)	(None, 7, 7, 512)	20024384
flatten_1 (Flatten)	(None, 25088)	0
dense_1 (Dense)	(None, 256)	6422784
dense_2 (Dense)	(None, 18)	4626

Total params: 26,451,794
Trainable params: 26,451,794
Non-trainable params: 0

Representation of the VGG19 Model as used:

Layer (type)	Output Shape	Param #
input_1 (InputLayer)	(None, 224, 224, 3)	0
block1_conv1 (Conv2D)	(None, 224, 224, 64)	1792
block1_conv2 (Conv2D)	(None, 224, 224, 64)	36928
block1_pool (MaxPooling2D)	(None, 112, 112, 64)	0
block2_conv1 (Conv2D)	(None, 112, 112, 128)	73856
block2_conv2 (Conv2D)	(None, 112, 112, 128)	147584
block2_pool (MaxPooling2D)	(None, 56, 56, 128)	0
block3_conv1 (Conv2D)	(None, 56, 56, 256)	295168
block3_conv2 (Conv2D)	(None, 56, 56, 256)	590080
block3_conv3 (Conv2D)	(None, 56, 56, 256)	590080
block3_conv4 (Conv2D)	(None, 56, 56, 256)	590080
block3_pool (MaxPooling2D)	(None, 28, 28, 256)	0
block4_conv1 (Conv2D)	(None, 28, 28, 512)	1180160
block4_conv2 (Conv2D)	(None, 28, 28, 512)	2359808
block4_conv3 (Conv2D)	(None, 28, 28, 512)	2359808
block4_conv4 (Conv2D)	(None, 28, 28, 512)	2359808
block4_pool (MaxPooling2D)	(None, 14, 14, 512)	0
block5_conv1 (Conv2D)	(None, 14, 14, 512)	2359808
block5_conv2 (Conv2D)	(None, 14, 14, 512)	2359808
block5_conv3 (Conv2D)	(None, 14, 14, 512)	2359808
block5_conv4 (Conv2D)	(None, 14, 14, 512)	2359808
block5_pool (MaxPooling2D)	(None, 7, 7, 512)	0

Total params: 20,024,384
Trainable params: 20,024,384
Non-trainable params: 0
